# Coping Strategies for Oral Health Problems by People with Schizophrenia

**DOI:** 10.1515/tnsci-2019-0033

**Published:** 2019-08-07

**Authors:** Francesca Siu Paredes, Nathalie Rude, Sahar Moussa-Badran, Jean-François Pelletier, Corinne Rat, Frederic Denis

**Affiliations:** 1 Université Champagne Ardenne. Faculté d’Odontologie de Reims, 51100 Reims, France; 2 EA 481 Integrative Neurosciences and Clinical, University Hospital of Besançon, F-25000 Besançon, France; 3 Department of Psychiatry, Montreal University, Yale Program for Recovery and Community Health, Montreal, Canada; 4 Clinical research unit, La Chartreuse psychiatric center, Dijon, France; 5 EA 75-05 Education, Ethique, Santé, Université de Tours, Faculté de Médecine, 37032 Tours, France; 6 Université de Nantes, Faculté d’Odontologie de Nantes, Nantes, France

**Keywords:** Oral health quality of life, schizophrenia, Coping strategies, Mental Health, Oral Health

## Abstract

**Background:**

Persons with schizophrenia are particularity susceptible to poor oral health. Symptoms of schizophrenia often affect oral health behaviors and lifestyle. The aim was to explore coping strategies used by people with schizophrenia in oral health in order to understand and to best involve them in the management of their own oral health in daily life.

**Materials and methods:**

This is systematic review reported in accordance with the Preferred Reporting Items for Systematic Reviews and Meta-Analyses (PRISMA) statements. We included cross-sectional and longitudinal quantitative and qualitative studies that 1) examined coping strategies regarding oral health in persons with schizophrenia or 2) examined coping strategies were used in dental care. We included studies conducted with at least one PWS aged 18 years old more and without restriction on sex, socioeconomic status, or language.

**Results:**

The 8 studies included suggest that coping strategies depends on complex translation processes that can be either personal (e.g., psychological symptomatology, neuropsychological functioning to adversely affect hope, self-esteem, self-stigma, self-determination, sense of coherence, and resilience) and/or environmental factors (e.g., peer support and efficacy of rehabilitations programs). We further identified that the main factor influencing coping strategies was dental stress situation.

**Conclusions:**

This review suggests that coping strategies play a crucial role in the recovery process for oral health of PWS. Translation processes in oral health should be more explored in the future to clarify the capacity of PWS to cope with essential self-care in oral health on daily life.

## Background

People with severe mental illness (SMI) like schizophrenia have a life expectancy that is 20 years lower than the general population [1]. Studies show that people with SMI have at least one associated somatic condition, including cardiovascular, gastrointestinal, respiratory, neoplastic, infectious, endocrine, and oral disorders. About half of these comorbidities are undiagnosed [2, 3, 4, 5, 6, 7]. Schizophrenia affects 0.7% and 1% of the world population and persons with schizophrenia (PWS) are particularity susceptible to a large number of somatic medical problems [1]. PWS often find obstacles in health care management such as inadequate pain management with devasting impacts of the exacerbations of mental illness and stability of psychiatric symptoms. Side effects from psychiatric medications are often not enough taken into consideration (e.g., weight gain, metabolic disturbances, poor oral health) with repercussions for non-adherence to psychotic treatments. Overall, these problems are obstacles to social support and recovery [8].

Mental disorders and in particular negative symptoms often affect both health behaviors and lifestyle that may lead to poor oral health and dental diseases [9]. For instance, side effects due to medications to manage schizophrenia, antipsychotics induce hyposalivation (xerostomia) or hypersalivation (clozapine) and/or oral dyskinesias with first-generation antipsychotics [10], excessive smoking [11], neglect of oral hygiene, carbohydrate rich diet [12], and use alcohol and illicit drugs [13]. Furthermore, PWS often experience social disadvantages, which in turn may affect their ability and desire to perform preventive oral hygiene [14]. For example, psychotic symptoms such as hallucinations and delusions that are often triggered or worsened by stress, can create an obstacle to dental treatment because procedures involved in those treatments may produce stressful and hence aversive stimulations [15].

Coping strategies are conscious efforts used by individuals to solve problems or needs in the daily life. The coping strategies were associated with both subjective (i.e., self-esteem and hopelessness) and objective (i.e., symptom severity) domains of recovery [17]. The existing literature shows that the effectiveness of coping strategies depends on the type of symptoms of the mental illness like stress, the individual and the circumstances [16].

In this case, understanding an individual’s physiological reaction to a stressor, or individual’s perception of a stress situation should also be considered in the schizophrenia context and oral health care. According to the transactional stress model of Lazarus and Folkman [18], the impact of a stressor is determined by one’s ability to cope with the situation, which in turn is related to the availability of various coping resources [18,19]. PWS also tend to have pervasive cognitive (memory and recall), suffer from personality alterations, self-esteem and social dysfunctions, both of this put these individuals at risk becoming isolated and victimized, reducing the effectiveness of learning new strategies to improve their health [20].

In summary, clinical indicators or measurement in oral health related quality

of life are insufficient to explain why PWS rarely consulted a dentist, are more likely to delay seeking care, and are less likely to adhere or receive adequate treatment than general population. Investigating out what’s coping strategies PWS used in daily life for taking as example a dentist appointment, brushing their teeth every day, or not, is an issue that warrants being addressed for better understanding why PWS don’t use healthcare system and good practices in oral health. We thus hypothesized that coping strategies in the context of schizophrenia plays a crucial role in the recovery processes of oral health when the person must deal with impediments to fulfil their goals

### Aim

To explore coping strategies used by PWS in oral health in order to more understand and to best involve them in the management of their own oral health in daily life.

## Methods

### Protocol and registration

Neither a review registration nor a review protocol was completed. This systematic review is reported in accordance with Cochrane Handbook [21] and the Preferred Reporting Items for Systematic Reviews and Meta-Analyses (PRISMA) statements for reporting systematic reviews of health sciences [22].

### Eligibility criteria

Based on the Participants–Intervention– Comparison–Outcome–Study (PICOS) method, we included cross-sectional and longitudinal quantitative and qualitative studies that1) examined coping strategies regarding oral health in PWS or 2) examined coping strategies were used in dental care. We included studies conducted with at least one PWS aged 18 years old more and without restriction on sex, socioeconomic status, or language. We excluded conference, abstracts, reviews and editorials.

### Data sources and search strategy

A comprehensive search was conducted up to December 15, 2018 by using systematic database searches of PUBMED (provided by National Center for Biotechnology Information, Medicine), PsycINFO (provided by EBSCOhost), SCOPUS (provided by Elsevier), LILACS (provided by BIREME-PAHO-WHO), GREY Literature Report (provided by The New York Academy Medicine) and Cochrane Library (provided by WILEY). The search strategy was developed with the assistance of a specialized health sciences librarian at the University of Reims, France.

First, we established the search terms on PubMed: (“Oral Health”[Mesh]) OR “Schizophrenia/ rehabilitation”[Mesh]) AND “Adaptation, Psychological”[Mesh]AND “Resilience”). The search for PsycINFO, SCOPUS, LILACS, GREY Literature Report and Cochrane Library were built of TITLE-ABS-KEY (schizophrenia) OR TITLE-ABS-KEY (oral AND health) AND TITLE-ABS-KEY (coping) OR TITLE-ABS-KEY (adaptation AND psychological) AND TITLE-ABS-KEY (resilience) AND TITLE-ABS-KEY (rehabilitation)).

Manual screening was then completed by searching through bibliographies and reference lists of the included papers to determine potential papers that were not found in the electronic search. Finally, a grey literature search was conducted by using Google Scholar and Google search engine.

### Study selection

Two reviewers (FSP and FD) independently screened the list of titles and abstracts to identify the potentially relevant papers based on the inclusion criteria. If the abstracts were judged to contain insufficient information, then the full studies were reviewed to decide whether they should be included based on the selection criteria. When a discrepancy in the selection decision occurred, the two reviewers engaged in discussion until a consensus was reached. If needed, a third reviewer (NR) resolved the possible conflicts concerning eligibility.

### Data extraction and data items

Two reviewers (FSP and FD) independently extracted data from the selected papers on the following items: Coping/adaptation, psychological, resilience, schizophrenia/ rehabilitation and oral health.

### Risk of bias in individual studies

Two reviewers (FSP and FD) independently assessed the methodological quality of the selected studies by using The Newcastle-Ottawa Scale [23] for cohort, cross-sectional, and case control studies; and the Critical Appraisal Skills Programme (CASP) checklist for qualitative studies [24].

## Results

### Study inclusion

**Figure 1:**
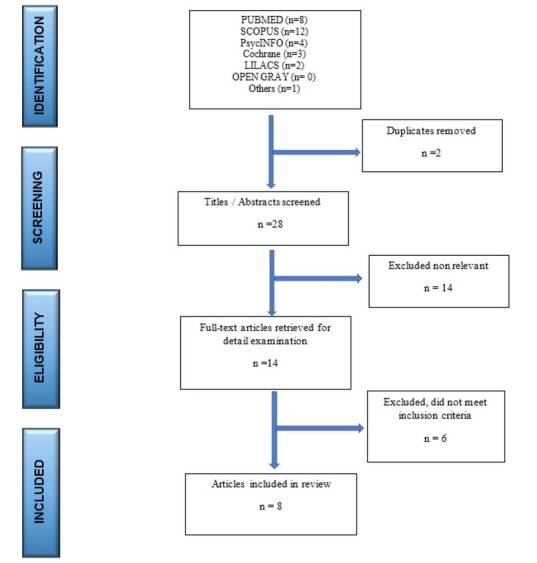
PRISMA flowchart of the identification process of included studies

An initial search identified 28 studies. After checking the title and reading the abstract 20 studies were excluded based on inclusion and exclusion criteria, and 8 studies were included in the review. Nothing else was found in grey literature. The PRISMA flow diagram that illustrates the search process [22] is presented in Figure 1.

### Characteristics of included studies

Of the 8 studies, all were qualitative and were written in English. These studies were conducted in Brazil (n = 1), Sweden (n = 1), China (n = 1), Romania (n = 1), Australia (n = 2), Taiwan (n = 1), and Switzerland (n = 1). A detailed description of the included studies is presented in Table 1.

**Table 1:** Factor’s influencing coping strategies

Author and year	Country	Participants	Evaluation	Factor’s influencing coping
[45]. Kao YC et al., (2017)	Taiwan	170 community-dwelling patients with schizophrenia	* Beck Cognitive Insight Scale (BCIS) The ISMI scale Self-esteem Scale (RSES) The COPE scale Positive and Negative Syndrome Scale (PANSS)	Self-reflection Self-stigma/ self-reflection Self-esteem, Psychotic symptomatology
[28]. Kelly A and Allott (2015)	Australia	34 with first-episode psychosis (FEP) and 26 healthy controls (HC)	** Brief Psychiatric Rating Scale (BPRS) Scale for the Assessment of Negative Symptoms (SANS) Hamilton Depression Rating Scale (HAMD) Anxiety Rating Scale (HAMA)	Neuropsychological functioning Stress
			Coping Inventory for Stressful Situations (CISS) Perceived Stress Scale (PSS) Neuropsychological assessment The Wide Range Achievement Test-Third Edition (WRAT-3)	
[31]. Martins AB et al., (2011)	Brazil	496 community dwelling adults aged 64 and older	**Resilience Questionnaire (RS) Structured questionnaire for sociodemographic information, health history, and health perceptions.	Sociodemographic health variables Resilience Health behaviors
[35]. Lindmark U et al., (2011)	Sweden	910 individuals aged 20, 30, 40, 50, 60, 70 and 80 years	**Swedish version of Antonovsky’s short version of the orientation to life / Sense of Coherence (SOC)	Sense of coherence
[39]. Chiu MY et al., (2009)	Chine	204 aged 18–60, with schizophrenia	**Resilience Scale (RS) Making Decision Empowerment scale (MDES) Self-Care Agency Scale (ESCA) Mastery Scale (MS) Adult State Hope Scale (ASHS) Support Scale of the Medical Outcome Study Social Support Survey–Chinese version (EISS-MOS-SSS-C) Schizophrenia Quality of Life Scale (SQLS) World Health Organization Spirituality Religion and Personal Belief Scale–Hong Kong version (WHO-SRPBHK).	Resilience Enporwement Sense of personal responsibility and self-determination Hope Peer support Psychological symptoms Spirituality
[40]. Dumitrescu AL et al., (2009)	Romania	198 first-year medical students	**Resilience Scale (RS)	Resilience Hope
[60]. Borras L et al., (2007)	Switzerland	103 stabilized patients with schizophrenia	*Religious Coping Index	Religiosity
[44]. Andresen R et al., (2006)	Australia	94 Persons with schizophrenia	**Recovery Assessment Scale (RAS) Mental Health Recovery Measure (MHRM)	Self-esteem Self-orientation Empowerment Hope Meaning Responsibility

*Qualitative method ** Quantitative method

### Synthesis of results

Due to the heterogeneity of the included studies, findings were evaluated in a descriptive manner. Differents thematic emerged:

- Neuropsychological functioning,
- Resilience,
- Sense of coherence,
- Sense and hope,
- Sense of personal responsibility and self-determination,
- Self-esteem, self confidence, stigmatisation,
- Stress,
- Social support/peer support
- Religious and spirituallity

### Neuropsychological functioning

To date, there have been relatively few studies aiming at establishing the relationship between neuropsychological functioning and coping strategies in schizophrenia. Nicholson et al. , suggested that neuropsychological deficits may interfere with the ability of individuals with schizophrenia to access effective coping skills or to use these mechanisms in a flexible, adaptive manner [25]. It is possible that problem solving skills, planning, abstract thinking, and the ability to access stored knowledge and strategies are the necessary cognitive prerequisites for engaging in activities that help people to redefine their lives and cope with the stressors associated with having a mental illness [26,27]. Kelly et al. 2015, showed that, coping style (specifically emotion-focused) was a more consistent predictor of perceived stress in the healthy control group than with persons with experience of a first-episode psychosis. This suggests that neuropsychological functioning (cognitive deficits in PWS) impact the flexibility with which individuals are able to select and apply a variety of coping strategies, as well as limit the use of active coping [28].

### Resilience

According to Grotberg et al., the resilience concept may play a role as a potentiating agent in adaptation to a problem, like tooth loss for example. Resilient behavior is associated with better adaptive abilities (such as coping) when facing adversities and incorporates interactions between diverse factors [29].

Resilience is a dynamic concept indicating that some individuals have a relatively good outcomes despite having experienced serious stresses or personal problems with a high resilience level. They also have better results in daily life than that of other individuals who suffered the same experiences [30]. A study of older adults reported the relationship between adaptive strategies following adverse events (tooth loss) and a positive oral health self-perceived. This suggests that a person with high resilience can cope with a negative fonctional evenement in a acceptable manner [31].

### Sense of coherence

Antonovsky et al., [32] introduced the concepts of sense of coherence (SOC) and general resistance resources (GRRs). Their study claims that peoples’ life orientation will have an impact on health and try to find the solution to the salutogenic question : why some people, regardless of major stressful situations and severe hardships, stay healthy, and use coping strategies while others do not ?

The SOC refers to an enduring attitude and measures how people view life and identify and use their GRRs (e.g. self‐esteem, intelligence) in stressful situations. A person with a strong SOC has many different GRRs and more importantly the ability to use these resources in a healthy direction (i.e. coping health behavior). Previous studies have demonstrated a strong relationship between SOC and dimensions of general health such as mental health [33,34], well-being and quality of life [33], and a weaker relationship between SOC and physical health [33]. Elias et al., showed that individuals with a strong or moderate SOC had significantly fewer oral health-related problems and thereby better oral health-related quality of life [35].

### Sense of hope

According to Snyder et al., hope is related to the life goals and expectations of a positive outcome of people’s own effort [36,37]. This concept of hope includes emotion, motivation, behavior and cognition [38]. Hope is an anticipation of a future which is good and based upon mutuality (relationships with others), self-steem competence, coping strategies, psychological well-being, purpose and meaning in life, as well as a sense of ‘’the possible’’. The basic level of hope remains relatively stable over time, which makes it resemble a personality trait. In PWS, the relationships among hope, depression, and self-stigma, sense of personal responsibility and self-determination are interconnected [39,40,41]. This relationship is illustrated with two adaptive tasks, which are common across situations that threaten physical or psychological well-being, managing uncertainty and coping with a changing reality. Coping fosters hope when it is at low ebb as well as ways in which hope fosters and sustains coping over the long term [42].

### Sense of personal responsibility and self-determination

Neither PWS nor their families have been involved in decision-making on dental health services, and they continue to be at risk of social exclusion and discrimination in their oral health care [43]. The experience of oral health for PWS can have lasting effects on a person’s sense of identity and self-worth the internalization of stigma. There is also evidence that the lack of influence or control on their care can lead to poor health outcomes. Conversely, the ability to exercise control and influence, even when high stress is present, can act as a protective factor against levels of disease risk. Powerlessness has emerged as a key risk factor in the etiology of disease, and evidences from a number of different fields suggest that empowerment is not just a set of values but also leads to recovery. Empowerment includes increased emotional wellbeing, independence in decision making, motivation to participate in health care and take care himselve and more effective coping strategies [39,44].

### Self-esteem, self confidence, stigmatisation

Self-esteem is an individual (emotional) evaluation of people’s own worth, so encompasses an implicit judgment of people’s ability to face life’s challenges (problem solving) that affects how you perceive stressors and deal with them (coping process). The adaptative coping strategies, were positively related to stigma resistance, self-stigma, self-esteem, self-reflection, and coping was significantly associated with higher stigma resistance [45]. The relationship between oral and general health is complex, particularly in PWS. Oral diseases affect the patient’s quality of life through social and psychological impacts such as the deterioration of the aesthetic of their smile, which leads to lower their self-esteem and self-confidence [46,47].

### Stress

Individuals with schizophrenia often report and demonstrate significant difficulty coping with life stresses [48,49] compared to people without a mental illness. PWS often employ a more limited range of coping strategies, which are characterized by a preference for avoidance and passive coping rather than help seeking or active problem-solving approaches [50,51].

Stress is a rather complex concept. However, effective coping enables people to be engaged in activities that may involve stress, such as dentist visits, which is one reason for their infrequent dentist visits [52]. Stress is subjective both in the measurement of severity and experience. The way in which individuals perceive and interpret stressors may vary greatly [53].

Two processes emerged, the perception of the nature and degree of risk divided into “threat” and “challenge” evaluations and the perception of resources or abilities to cope with the stressor.

Kelly et al., suggested that although coping style and neuropsychological functioning are important predictors of perceived stress by PWS, additional factors need to be considered such as social support, expressed emotion, self-esteem, resilience and self-efficacy as trait negative affectivity is significantly related to coping styles and stress reactivity in schizophrenia [28].

### Social Support /Peer support

Social supports develop self-esteem based on the feedback of individuals from the environment as a result of social interactions [54,55]. A personal support network is essential for coping, building and maintaining resilience. Encouragement and support from these relationships is extremely effective in helping people work through stressful periods. Peers can be a vitally important component of support network, because they are likely to be experiencing similar stresses [39].

### Religious and spirituallity

Religious coping is multidimensional and refers to functionally oriented expressions of religion in times of stress. Religious coping is operationally defined as “the use of religious beliefs or behaviors to facilitate problem-solving to prevent or alleviate the negative emotional consequences of stressful life circumstances” [56]. Carver et al. [57] suggested that the value of positive reinterpretations is that it not only reduces distress, but also can be used to reappraise a stressful situation and see it more positively.

Studies revealed that adaptive coping strategies were significantly associated with religious and spirituallity as resource of finding meaning and hope were identified as a key component of the process of psychological recovery [58,59,60].

**Figure 2:**
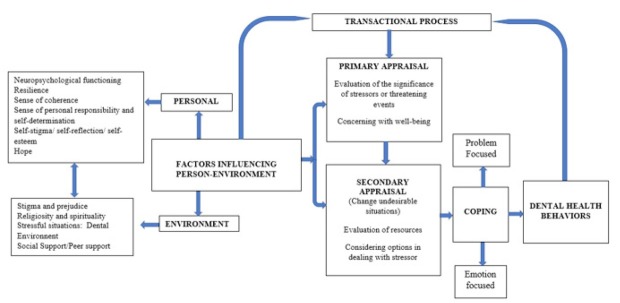
Metacognitive process in oral health for person with schizophrenia

All of this these metacognitive processes are summarized in Figure 2.

## Discussion

The aim of this review was to explore coping strategies used by PWS in oral health in order to more understand and to best involve them in the management of their own oral health in daily life.

This work suggests that dental health behaviors for PWS were associated with many personal factors (e.g. psychological symptomatology, neuropsychological functioning, self-esteem, self-stigma, self-determination, sense of coherence, and resilience), The peer support and efficacy of rehabilitations programs have been impacting to dental stressful situations, Studies described complex interactions between sense of coherence, resilience, stigma-resistance, self-resistance and, sociocultural factors like religiosity, spirituality, hope, stressful situations, the social support and peer support.

Our work highlights that coping strategies were considered as the evaluation of a transaction between the people and their environment and was important to cope oral health problems. In other words, coping strategies are conscious efforts used to solve problems or to fulfil needs in daily life. Coping strategies are associated with both subjective (self-esteem and hopelessness) and objective (symptom severity) domains of recovery [17]. The effectiveness of the coping effort appears to depend on the symptoms of the mental illness, such as stress; the individual and the circumstances [16]. Conversely, the use of effective coping strategies, emotional wellbeing, independence in decision making, motivation contribute participation in health care, even when high stress is present [39,44].

To our knowledge, no study has investigated specifically the strategies to cope the oral health problems that schizophrenic patients go through. However, some studies described that PWS neglect their self-care and have high rates of physical ill-health, including oral health [61,62], poor lifestyle oral health behaviors (diet rich in sugars, use of psychoactive substances such as tobacco, and inadequate oral hygiene) [63,64]. We have observed difficult relationships with professional caregivers (such as fear of mental illness and lack of training) and the health system in general (including difficulties in gaining access to private practice, environment, and cost) are additional obstacles contributing to deficient somatic care [6].

Considering the stakes, a social support and/or peer support is essential to promote oral health for PWS and learning programs in oral health should be specific for PWS. This is maybe why programs based on knowledge in the general population and transposed for persons with severe mental disorders, have not proven to be very effective. The specific profile of PWS must be taken into account [44]. Indeed, schizophrenia interferes with a person’s ability to manage their emotions and make decisions in daily life [66,67]. Coping strategies play a crucial role in the recovery process in oral health of PWS.

A better understanding of the interest or disinterest in the act of self-care is needed to help PWS to improve their oral health quality of life. Studies revelated that Motivational Interviewing Program improved service user’s knowledge and the ability to maintain a better oral health care, self-regulation and plaques index scores (possibly mediated by improvement a dental hygiene behavior, such as improved tooth brushing) [68,69].

## Conclussions

This is the first study to examine the appraisal and coping processes associated with oral health in PWS. We highlighted that coping strategies for PWS in oral health were considered as the evaluation of a transaction between people and their environment where certain perceptive, emotional or behavioral processes can cope with stressful events. Although, coping strategies play a crucial role in the recovery process of PWS the mechanisms of this involvement should more specialty explored in the future to clarified the capacity of PWS to cope with essential self-care on daily life for good oral health related quality of life.

These results could be used to implement research in building oral health prevention programs taking into account a metacognitive approach and no only focused in oral health pathologies
